# Proteome and Transcriptome Analysis of the Antioxidant Mechanism in Chicken Regulated by Eucalyptus Leaf Polyphenols Extract

**DOI:** 10.1155/2020/1384907

**Published:** 2020-06-14

**Authors:** Wei Li, Ze-qi He, Xiao-Ying Zhang, Yun-Jiao Chen, Jian-Jun Zuo, Yong Cao

**Affiliations:** ^1^College of Food Science, South China Agricultural University, Guangdong Provincial Key Laboratory of Nutraceuticals and Functional Foods, Guangdong Research Center for Engineering Technology in Bioactive Natural Products, Guangzhou 510642, China; ^2^College of Animal Science, South China Agriculture University, Guangzhou 510642, China

## Abstract

Eucalyptus leaf polyphenols extract (EPE) has been proved to have various bioactivities, but few reports focus on its antioxidant mechanism *in vivo*. The purpose of this study was to elucidate the effect and mechanism of EPE dietary supplements on antioxidant capacity in chicken. A total of 216 chickens were randomly selected for a 40-day experiment. Four treatment groups received diets including the control diet only, the control diet + low EPE (0.6 g/kg), the control diet + moderate EPE (0.9 g/kg), and the control diet + high EPE (1.2 g/kg). Compared with control group, the glutathione peroxidase (GSH-Px) activity and glutathione (GSH) content in the breast muscle of the moderate EPE treatment group was significantly higher (*p* < 0.05), while the malonaldehyde (MDA) content in the moderate EPE group was reduced (*p* < 0.05). Moreover, proteomic and transcriptomic analyses of the breast muscle revealed that glutathione metabolism and the peroxisome were the two crucial metabolic pathways responsible for increased antioxidant capacity of the muscle. Accordingly, nine candidate genes and two candidate proteins were identified related to improved antioxidant status induced by EPE supplements. This research provides new insights into the molecular mechanism of antioxidant capacity in chickens treated with EPE dietary supplements.

## 1. Introduction

The genus *Eucalyptus* comprises over 900 species of economically valuable plants endemic to Australia [1, 2]. In China, the eucalyptus plantation area reached 4.5 million hm^2^ by the end of 2016 [3]. There is a growing need to develop alternative uses for eucalyptus products, and eucalyptus plants have attracted broad interest among researchers to investigate their functionality and potential applications [4–6]. Eucalyptus leaves are known to contain numerous bioactive substances including, but not limited to, terpenoids, tannins, flavonoids, and phloroglucinol derivatives, and eucalyptus has also been shown to function as antioxidants [7, 8]. Accordingly, bioactive components derived from eucalyptus leaves and their functions for use in the food industry are widely researched, with many studies focused on their antioxidant capacity [9–11].

Recent technological advances in proteomics and transcriptomics have substantially improved the understanding of how variation in proteins and genes impact living organisms [12–15]. In particular, these methods may be used to elucidate the molecular mechanism of antioxidant capacity. Using proteomic techniques, Wang et al. [16] discovered that broiler chickens given albusin B supplements could upregulate the expression of GST, Prdx6, PPIA, alfatoxin aldehyde reductase, and superoxide dismutase to improve antioxidant defense.

Our previous study revealed that EPE exhibited a strong antioxidant activity in chemical-based and cellular-based assay [7]. Moreover, EPE treatment could protect acute-induced oxidative damage by improving antioxidant enzymes (GSH-Px, T-SOD) in chicken [7]. Several researches also reported that diet with eucalyptus leaves extract significantly improves antioxidant status [17, 18]. Eucalyptus leaves extracts with immense biomass and great antioxidant potential benefited to improve growth performance and health status of broilers, which could be useful for the poultry industry [19, 20]. However, few studies have investigated the antioxidant mechanism of EPE in chicken. Therefore, this study was performed to reveal EPE dietary supplements on the antioxidant mechanism of chicken using proteomic and transcriptomic analysis.

## 2. Materials and Methods

### 2.1. Experimental Material

All the Huiyang beard chickens used in this study were provided by Xingtai Modern Agricultural Limited Company of Huizhou in Guangdong, China.

EPE: Eucalyptus leaves (*Eucalyptus grandis* × *Eucalyptus urophylla* GL9) were picked in October in Zhanjiang and air-dried naturally (7~9 d, 28 ± 2°C, moisture content 15.53 ± 2.11%). The eucalyptus leaves were extracted by 70% ethanol solvent (*v*/*v*) at 75°C. And then the extraction solution was vacuum-concentrated and spray-dried (the yield of EPE was 25.78 ± 2.03%), the EPE was stored at 4°C for the subsequent experiment.

The total polyphenol content in EPE was determined using the Folin-phenol method [21]. Additionally, the EPE was analyzed using high-performance liquid chromatography (HPLC) with a Diamonsil C18 column (250 × 4.6 mm, 5 *μ*m, Diamonsil, China). EPE was reconstituted in buffer A (H_2_O) and loaded onto the column. It was then eluted using gradient buffer B (Methanol) in the following order: 10% at 0 min and 10~90% at 60 min. The flow rate was 1 mL/min and the injection volume was 10 *μ*L. The column temperature was 37°C and a detection wavelength of 270 nm.

### 2.2. Animal Experimental Design

A total of 216 chickens (90 days old) were randomly assigned to one of four treatments (6 pens/treatment and 6 chicken/pen), and the duration of the trial was 40 days (from 90 to 130 days). Four treatment groups received diets including control diet only (Table 1), low EPE (control diet+0.6 g/kg EPE), moderate EPE (control diet+0.9 g/kg EPE), and high EPE (control diet+1.2 g/kg EPE). The experimental design and procedures were approved by following the requirements of the Regulations for the Administration of Affairs Concerning Experimental Animals of China.

### 2.3. Experimental Basic Fodder and Management

Ingredients and nutrient compositions of the diets are shown in Table 1, and the diets were used throughout the whole experimental period. The chickens were raised in cages in a controlled environment and had free access to food and drinking water. The temperature in the house was 24~29°C, and the relative humidity was 60~78%. Pens were routinely disinfected to maintain appropriate standards of cleanliness. All animal procedures were conducted under the protocol (SCAU-AEC-2010-0416) approved by the Animal Ethics Committee of South China Agricultural University.

### 2.4. Sample Collection

After 40 days, 12 chickens were randomly selected from each treatment group (2 broiler chickens from each replication), and blood samples were drawn from the neck vein with a sterile syringe. The blood was centrifuged at 3000 × g for 15 min to obtain serum samples, and they were stored at -80°C until analysis. Chickens were then euthanized, and the whole breast muscle tissues were preserved in liquid nitrogen.

### 2.5. Antioxidant Activity in Serum and Muscle Tissue

About 0.25 g muscle tissue was mixed with 9 times (*m*/*v*) 0.9% physiological saline, and then, homogenized with a tissue grinder (Lawson, Japan) at 4°C. After being centrifuged for 10 min at 4000 r/min (Eppendorf, Germany), the supernatant was used for subsequent analysis. The activities of total superoxide dismutase (T-SOD, A001-1-2), GSH-Px (A005-1-2), total antioxidant capacity (T-AOC, A015-1-2), the contents of malondialdehyde (MDA, A003-1-2), and GSH (A061-1-1) in serum and breast muscle were determined spectrophotometrically using the commercial kits obtained from Nanjing Jiancheng Institute of Bioengineering (Nanjing, Jiangsu, China) [22]. The quantities of T-SOD, GSH-Px, and T-AOC are expressed as units (U) per milligram of protein. The MDA and GSH content are expressed as nanomoles per milligram of protein and milligram per gram of protein, respectively.

### 2.6. Screening and Annotation of Differentially Expressed Genes (DEGs) in Muscle Induced by EPE Dietary Supplements in Chicken Based on RNA-Seq Techniques

Based on the evaluation of antioxidant activity in this study and a previous study [23], the moderate EPE treatment group tended to exhibit higher antioxidant effects compared with the other group. Therefore, breast muscles were obtained from the control group and the moderate EPE treatment group (0.9 g/kg) for transcriptome sequencing, with two replicates per group [24].

#### 2.6.1. Total RNA Isolation, Labeling, and Sequencing

Total RNA was extracted from muscle samples. RNA concentration and purity were evaluated by Nanodrop 2000 (Thermo, USA), and RNA integrity was determined by 1% agarose gel electrophoresis. Then, mRNA was isolated from the total RNA using Oligo (dT) beads. Fragmentation buffer was used to randomly shear mRNA into 200 bp fragments. Using reverse transcriptase, random hexamers were added to synthesize a strand of cDNA from the mRNA template, followed by two-strand synthesis to form a stable double-stranded structure. The viscous end of the double-stranded cDNA structure was repaired using End Repair Mix (Enzymatics, USA), followed by the addition of an A base at the 3′ end to form the Y-form linker. The final library was sequenced on an Illumina Hiseq (Hokkaido System Science, Sapporo, Japan) [25, 26].

#### 2.6.2. Data Analysis and Bioinformatics of Genes

Chicken transcriptome sequencing was carried out using an Illumina sequencing platform (2 × 150 bp, 375 bp insert size). Quality control of the sequenced data was completed, and the transcriptome data were analyzed using an established bioinformatics method [27]. DEGs were calculated based on gene read count data by the edgeR software, and the screening criteria for DEGs were False discovery rate (FDR) < 0.05, *p* value < 0.05 and fold change (FC) > 2. Gene Ontology (GO) and Kyoto Encyclopedia of Genes and Genomes (KEGG) were used to annotate and enrich the DEGs.

### 2.7. Screening and Annotation of Differentially Expressed Proteins (DEPs) in Muscle Induced by EPE Dietary Supplements in Chicken Based on iTRAQ Techniques

The breast muscle was obtained from the control group and the moderate (0.9 g/kg) EPE group for proteomic analysis, with two replicates per group [28].

#### 2.7.1. Two-Dimensional Fluorescence Difference Gel Electrophoresis

About 15 mg of breast muscle was manually ground with liquid nitrogen, and then, 8 M urea containing 1% sodium dodecyl sulfate (Sinopharm Chemical Reagent Co., Ltd, China) and protease inhibitor were added in a 1 : 5 ratio. The solution was placed on ice in an ultrasound wave for 2 min and then centrifuged at 4°C for 20 min. The supernatant was taken to determine the protein concentration via gel electrophoresis. A 100 microgram aliquot of protein sample was dissolved with 100 *μ*l 8 M urea containing 1% sodium dodecyl sulfate, and then, 10 mM TCEP solution (Thermo, USA) was added to the sample and incubated at 37°C for 60 min. 40 mM iodoacetamide (Sigma, USA) was then added, and the solution was incubated at room temperature for 40 min. Precooled acetone (Sinopharm Chemical Reagent Co., Ltd, China) was added in a 6 : 1 ratio (acetone : sample). Samples were precipitated at -20°C for 4 h, followed by centrifugation at 10000 g for 20 min. Then, samples were digested overnight at 37°C with 100 *μ*L 100 mM triethylammonium bicarbonate buffer (TEAB, Sigma, USA), and trypsin was added according to a mass ratio of 1 : 25 (enzyme : protein) [29].

#### 2.7.2. Identification of DEPs

Muscle was digested with trypsin, and the peptide was dried with a vacuum pump and redissolved with a 0.4 M TEAB solution. The iTRAQ reagent (AB Sciex, USA) was added per 100 *μ*g of peptide, and samples were incubated for 2 h, followed by adding 50 *μ*L ultrapure water and incubating for 30 min. Each group of labeled product was mixed and dried. The peptide samples were reconstituted with UPLC loading buffer and separated by C18 column during the pH liquid phase.

The first-dimensional separation was performed using Waters' ACQUITY UPLC BEH C18 column (3 mm × 150 mm, 1.7 *μ*m, Waters, USA) with a flow rate of 400 *μ*L/min. The detection wavelength was 214 nm. The labeled peptides were reconstituted in buffer A (2% acetonitrile, pH 10.0) and loaded onto the column. The peptides were then eluted at 37°C using gradient buffer B (80% acetonitrile, pH 10.0) in the following order: 0% in 2 min, 0~3.8% in 15 min, 3.8~24% in 18 min, 24~30% in 3 min, 30~43% in 1 min, 43~100% in 1 min, 100~0% in 6 min, and 0% keeping for 20 min.

The second-dimensional separation was performed using Liquid-mass spectrometry. The chromatographic instrument was EASY-nLC 1200 (75 *μ*m × 25 cm, Thermo, USA), the mass spectrometer was Q-Exactive (Thermo, USA), and the data acquisition software was Thermo Xcalibur 4.0 (Thermo, USA). The samples were reconstituted in buffer A (2% acetonitrile with 0.1% formic acid) and loaded onto the column. The peptides were then eluted at 37°C using gradient buffer B (80% acetonitrile with 0.1% formic acid) in the following order: 0~5% in 1 min, 5~23% in 62 min, 23~48% in 25 min, 48~100% in 1 min, 100% keeping for 6 min, 100~0% in 5 min, and 0% keeping for 20 min. Mass spectrometry conditions were as follows: MS scan range (*m*/*z*) 350-1300, acquisition mode DDA; Top 20 (select the strongest signal in the parent ion 20 for secondary fragmentation); first-order mass spectrometry resolution of 70000, fragmentation HCD; Resolution 17500, dynamic exclusion time 18 s.

#### 2.7.3. Bioinformatic Analysis of Proteins

The GO analysis and the KEGG pathways analysis were implemented in KOBAS [30], and significance was evaluated using Fisher's exact test.

### 2.8. Statistical Analysis

Data were analyzed by one-way ANOVA (SPSS, 2010). Differences among treatment groups were evaluated using Duncan's multiple range tests, with a significance threshold of *p* < 0.05.

## 3. Results

### 3.1. Quality of EPE

**Figure 1:**
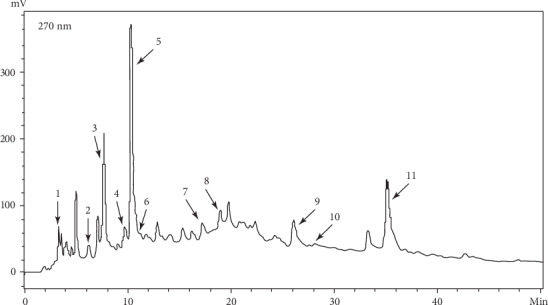
High-performance liquid chromatography of EPE. Note: 1 Gemin D; 2, 4 Pedunculagin; 3 Gallic acid; 5 Oenothein B; 6,7 TellimagrandinI; 8 Chlorongenic acid; 9 Ethyl gallate; 10 1,2,3,4,6-*O*-pentagalloylglucose; 11 Hyperoside.

According to the Folin-reagent method described by Wang et al. (Gallic acid as the equivalent polyphenol) [21], the total polyphenols content in EPE was 317.08 ± 16.49 mg/g. Further, HPLC of EPE (Figure 1) indicated that the main active substance was of comparable quality to the EPE reported in our previous research, based on the standard substance retention time [23].

### 3.2. Variation of Antioxidant Capacity in Serum

As shown in Table 2, the concentrations of GSH-Px, T-SOD, T-AOC, and MDA in serum were not significantly different among the treatment groups (*p* > 0.05). Compared with the control group, the T-SOD activity and T-AOC increased in the EPE treatment groups, and the GSH-Px activity in the moderate EPE group increased (0.9 g/kg) by 13.8% (*p* > 0.05).

### 3.3. Variation of Antioxidant Capacity in Muscle Tissue

As shown in Table 3, the T-SOD activity and T-AOC of the breast muscle in the moderate EPE (0.9 g/kg) and high EPE (1.2 g/kg) groups showed an increasing trend compared to the control group (*p* > 0.05). The GSH-Px activity of the breast muscle tissue in the moderate (0.9 g/kg) EPE group was 44.5%, higher than that of the control group (*p* < 0.05), and there was an upward trend in the low (0.6 g/kg) and high (1.2 g/kg) EPE groups (*p* > 0.05). The GSH content of breast muscle in the moderate and high EPE groups increased by 23.3% and 20.7% compared with the control group (*p* < 0.05). Additionally, MDA content in the breast muscle of the moderate (0.9 g/kg) EPE group was reduced by 25.4% (*p* < 0.05), though the other two groups did not show a significant change (*p* > 0.05).

### 3.4. Screening and Annotation of DEGs in Muscle Induced by EPE Dietary Supplements in Chicken Based on RNA-Seq Techniques

**Figure 2:**
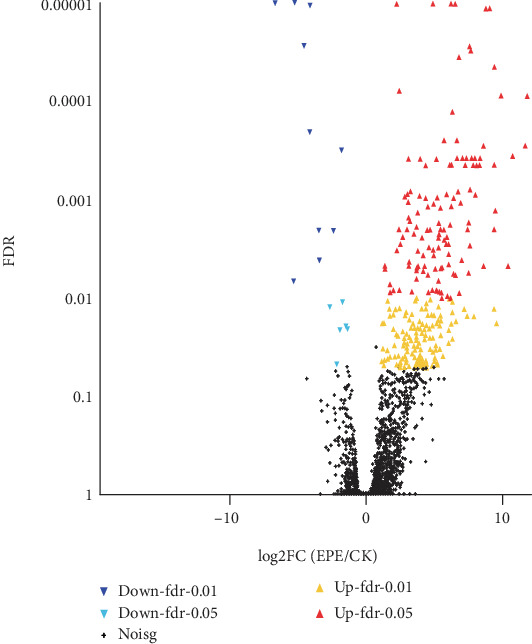
Volcano plots of DEGs. Note: differentially expressed genes (DEGs). The abscissa is the fold change of the gene's expression difference. The ordinate is the statistical test value of the difference in the amount of gene arrival, and the higher the *p* value, the more significant the difference in expression. Red dots indicate significantly upregulated genes, blue dots indicate significantly down-regulated genes, and black dots are nonsignificant differentially expressed genes.

To ensure high quality data, raw sequencing reads were filtered based on coverage, sequence saturation, the redundant distribution frequency map, and the distribution of sequencing reads across the chromosomes. A total of 14621 genes were identified as differentially regulated, of which 289 were significantly different between the EPE treatment group and the control group. The overall distribution of gene expression differences was visualized by a volcanic map (Figure 2).

#### 3.4.1. Gene Ontology Enrichment Analysis of the DEGs

**Figure 3:**
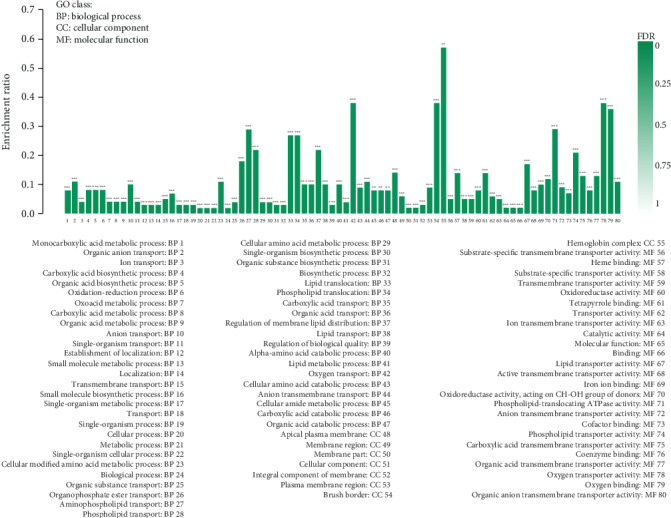
GO enrichment histogram for DEGs. Note: differentially expressed genes (DEGs). Each column in the figure is a GO term, and the abscissa text indicates the name and classification of the GO term. The height of the column, that is, the ordinate, indicates the enrichment rate. The color indicates the significance of enrichment, that is, FDR. The darker the color, the more significant the enrichment of the GO term, wherein the mark with FDR < 0.001 is ^∗∗∗^, the mark with FDR < 0.01 is ^∗∗^, and the mark with FDR < 0.05 is ^∗^, the right color gradient indicates the FDR size.

Gene Ontology (GO) enrichment analysis was used to annotate these DEGs by biological process, cellular component, and molecular function (Figure 3). Overall, 47 different biological processes were enriched, including oxygen transport, oxidation-reduction, aminophospholipid transport, phospholipid translocation, and lipid translocation. Cellular component analysis revealed that the apical plasma membrane, membrane region, membrane part, cellular component, integral component of membrane, plasma membrane region, brush border, hemoglobin complex, and substrate-specific transporter activity were significantly enriched. Additionally, oxygen binding, oxygen transporter activity, phospholipid-translocating ATPase activity, and other 22 molecular function items were identified by the molecular function analysis.

Based on the above GO enrichment analysis, glutathione metabolic process (biological process), glutathione transferase activity (molecular function), and peroxisome (cellular component) were the enriched GO terms most clearly associated with antioxidant capacity (Table 4). Overall, 4 antioxidant genes (gamma-glutamyltransferase 1, GGT1; microsomal glutathione S-transferase 1, MGST1; glutathione S-transferase alpha 4, GSTA4L; and glutathione S-transferase class-alpha, GSTAL1) in the EPE group were substantially enhanced in glutathione metabolic process relative to the control group. Similarly, three of these genes (MGST1, GSTA4L, and GSTAL1) were also associated with glutathione metabolic process and glutathione transferase activity, which play an important role in muscle antioxidant activity. Additionally, hydroxyacid oxidase 1 (HAO1), hydroxyacid oxidase 2 (HAO2), and bile acid-CoA: amino acid N-acyltransferase (BAAT) are peroxisome genes known to be involved in the oxidation of fatty acids, regulation of oxygen concentration, and decomposition of hydrogen peroxide to improve the antioxidant status in breast muscle.

#### 3.4.2. Kyoto Encyclopedia of Genes and Genomes (KEGG) Analysis of the DEGs

DEGs were enriched in 148 pathways in the KEGG enrichment analysis, and 38 KEGG pathways were identified to have significant changes. Based on the previous analysis of physiological indicators, the peroxisomes and the glutathione metabolism pathway were selected for further evaluation of the antioxidant capacity of the muscle by KEGG enrichment metabolic pathway analysis. Significant DEGs that were enriched in the peroxisomes and glutathione metabolism pathway showed some overlap with the genes related to antioxidant status identified by the GO enrichment analysis, including HAO1, HAO2, and GGT1 (Table 5).

### 3.5. Screening and Annotation of DEPs in Muscle Induced by EPE Dietary Supplements in Chicken Based on iTRAQ Techniques

#### 3.5.1. Identification and GO Analysis of DEPs Induced by EPE

**Figure 4:**
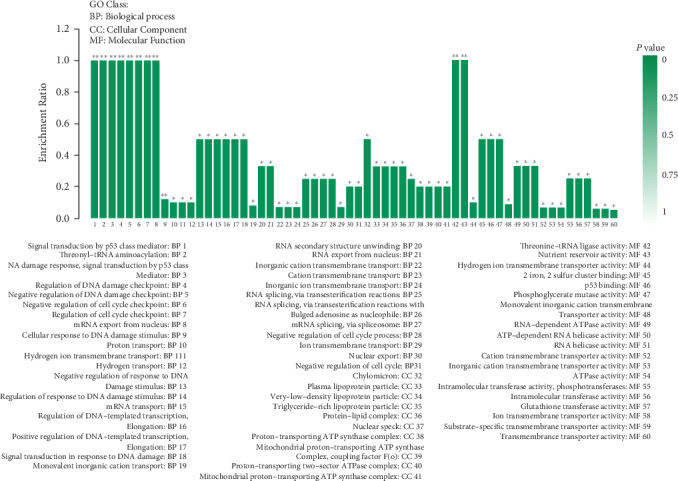
GO enrichment histogram for DEPs. Note: differentially expressed proteins (DEPs). Each column in the figure is a GO term, and the abscissa text indicates the name and classification of the GO. The height of the column, that is, the ordinate, indicates the enrichment rate. The color indicates the significance of enrichment, that is, FDR. The darker the color, the more significant the enrichment of the GO term, wherein the mark with FDR < 0.001 is ^∗∗∗^, the mark with FDR < 0.01 is ^∗∗^, and the mark with FDR < 0.05 is ^∗^, the right color gradient indicates the FDR size.

The total number of tested proteins was 1430, and there were 149 protein differences between the EPE group and the control group. According to the standard of differential protein screening, there were 14 significant differences in protein concentration (>1.2-fold change, *p* < 0.05), 10 of which were upregulated and 4 were downregulated (Table 6). These proteins were annotated (cellular components, molecular functions, and biological processes) by GO enrichment analysis (Figure 4). Biological process analysis revealed that most proteins were associated with 31 different GO terms, including p53 class mediator, threonyl-tRNA aminoacylation, and regulation of DNA damage checkpoint. When DEPs were annotated by the cell components, significantly enriched terms included chylomicron, plasma lipoprotein particle, very-lower-density lipoprotein particle, triglyceride-rich lipoprotein particle, protein-lipid complex, and five other cellular components terms. A total of five DEPS were annotated by molecular function, and there was significant enrichment of nineteen GO terms, including threonine-tRNA ligase activity, nutrient reservoir activity, p53 binding, and glutathione transferase activity.

#### 3.5.2. KEGG Pathway Analysis of the DEPs

DEPs were analyses by KEGG pathway analysis and the results indicated that cytochrome b-c1 complex subunit Rieske (Q5ZLR5), A0A0A0MQ61, and F1P372 were main proteins associated with oxidative phosphorylation, glutathione metabolism, aminoacyl-tRNA biosynthesis, metabolism of xenobiotics by cytochrome P450, etc. (Table 7).

### 3.6. Combined Analysis of Transcriptomics and Proteomics

**Figure 5:**
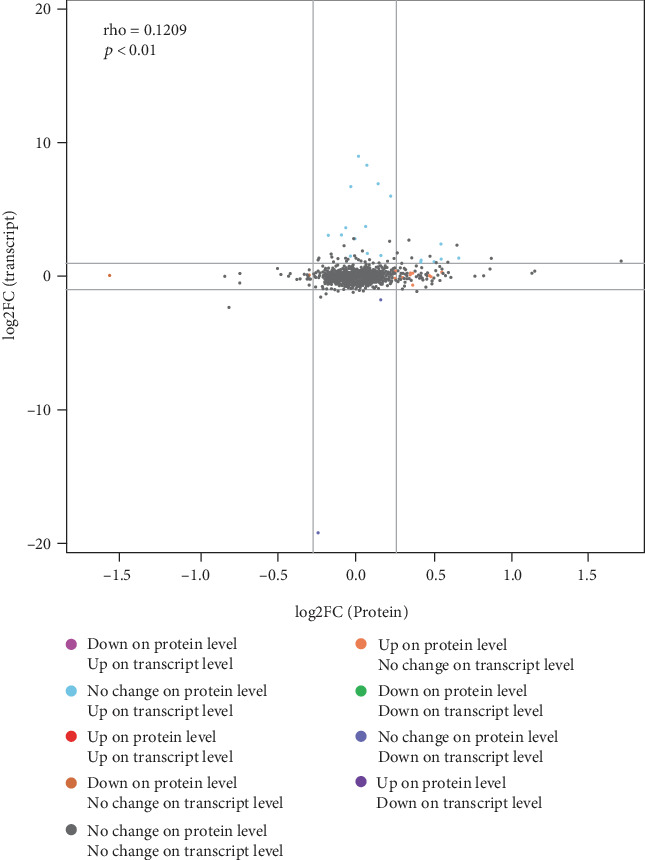
A scatter plots of the expression levels of all proteins and their associated transcripts in both groups. Note: the abscissa in the figure indicates the difference in the expression of the protein in the EPE treatment group and the control group, and the ordinate indicates the difference in the FPKM value of the corresponding transcript in the experimental group and the control group. Each point represents a protein and its associated transcript; in the upper left corner, rho represents the Pearson's correlation coefficient between the two omics, *p* represents the correlation test *p* value; when rho > 0, it is called the negative correlation; When rho < 0, it is called positive correlation; when rho = 0, it is called zero correlation, that is, there is no correlation; the larger the∣rho∣, the greater the correlation between the two omics.

**Figure 6:**
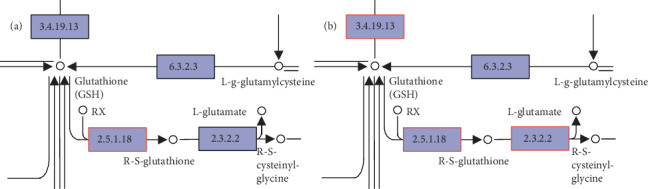
Upregulated protein in the proteome (a) and genes in the transcriptome (b) in glutathione metabolism. Note: the genes with red borders in the figure belong to the DEGs or DEPs detected by this sequencing, in which red represents the upregulated gene.

All proteins and their associated transcripts in both the transcriptomic and proteomic analyses were classified into nine categories (Figure 5). Of the 61 proteins and genes that were upregulated in the EPE group, the A0A0A0MQ61 protein and the glutathione S-transferase alpha-like 2 gene (GSTAL2) played a crucial role in enhancing antioxidant status. The glutathione metabolism in the transcriptomic analysis indicated that GGT1, MGST1, and GSTA4L were downstream genes regulated by glutathione, which correspond to the observed increase in GSH-Px activity in muscle tissue induced by EPE supplements. Accordingly, A0A0A0MQ61, the protein product of the glutathione S-transferase gene, was substantially upregulated in glutathione metabolism, which was in line with the expression of GGT1, MGST1, and GSTA4L (Figure 6). Therefore, EPE may enhance the antioxidant status of chicken by improving the activity of specific antioxidant proteins.

## 4. Discussion

Phytogenic feed additives have been gaining attention in improving the health status of the flocks [31, 32]. Our previous study revealed that diet with polyphenols obtained from eucalyptus leaves exhibited a positive effect on growth performance in laying hens [23]. In the present study, different concentrations of EPE in chicken diet did not exhibit significant positive or negative effect on chicken performance, including average daily gain, average daily feed take, and feed conversion rate (data not shown), which is in agreement with the report of Sedaghat and Torshizi [33], and might be due to that the chickens were in the adulthood.

Recently, natural polyphenols used in animal diets have been reported to build an integrated antioxidant system to prevent from damage led by free radicals [34–37]. This antioxidant capacity is assumed to result from the free radical-scavenging properties of phenolic compounds [38–40]. Our previous study reported that EPE effectively scavenged DPPH• and ABTS• free radicals *in vitro* [21]. In the present study, the antioxidant effects in serum were not obviously affected by the EPE supplement. However, an increasing trend in GSH-Px was observed with a higher concentration of the EPE diet, whereas the MDA content showed the opposite effects. This result was supported by the previous report that diet with 0.8 g/kg polyphenols from eucalyptus leaves increased the GSH-Px activity in serum of laying hen and an insignificant decrease in MDA was observed in quails supplement with eucalyptus leaves [23, 31]. Interestingly, our study revealed that EPE dietary supplements significantly improved GSH-Px activity and GSH content and decreased MDA content in breast muscle tissues. Higher concentration of the EPE diet led to higher GSH-Px activity, which is consistent with the report by Fathi et al. [31]. Similarly, MDA content can reflect the degree of lipid peroxidation; thereby, indirectly reflect cell damage and freshness of meat [41]. In agreement with our results, a diet with green tea extract led to a remarkably decreasing MDA content in meat tissue [42]. Polyphenols might be accumulated to exhibit antioxidant effects in meat tissues [35]. To explain the antioxidant capacity of polyphenols, it is crucial to understand how they are absorbed, metabolized, and eliminated from the body. It is stated that the intestinal utilization of polyphenols depended on their degree of polymerization and galloylation [43]. Monomeric and some oligomeric polyphenols tended to be easily absorbed at small intestine compared with the polymeric forms of polyphenols [44]. This suggested that monomeric and some oligomeric polyphenols in EPE, such as gallic acid, pedunculagin, hyperoside, and other compounds, could be absorbed and functioned as an antioxidant in chickens. This hypothesis was supported by Chamorro et al. that monomeric (catechin, epicatechin, gallic acid, and epicatechin-O-gallate) and dimeric (procyanidin B1 and procyanidin B2) catechins in grape pomace were easily digested and absorbed in chickens [45]. Overall, EPE dietary supplements are able to improve the antioxidant capacity of chicken.

The results of the transcriptomic analysis indicated that glutathione transferase activity, glutathione metabolic process, and the peroxisome were the three GO enrichment terms related to antioxidant activity. That is, several significantly upregulated antioxidant genes, such as GGT1, MGST1, GSTA4L, HAO1, and HAO2, are all believed to improve antioxidant status. Moreover, previous research indicated that GGT1 and PGDS play an important role in the synthesis of glutathione, and its upregulation exerts a prooxidant action [46]. Shinno et al. [47] reported that microsomal glutathione S-transferase 1 (MGST1) could be activated by gallic acid to protect the membrane against damage caused by oxidative stress. These results suggested that these antioxidant genes were vital to improve antioxidant status in chicken. Likewise, the KEGG pathway enrichment analysis also indicated that the peroxisomes and the glutathione metabolism pathway were the two crucial antioxidant pathways. Peroxisomes are rich in enzymes, mainly including oxidases, catalase, and peroxidase. Catalase, in particular, is known to protect cells by hydrolyzing the hydrogen peroxide generated in redox reactions [48, 49]. Additionally, several differentially expressed antioxidant-related genes were significantly enriched in the glutathione metabolism pathway, and the content of glutathione may be altered in cells as a result of EPE supplements. Glutathione is known to effectively scavenge free radicals and other reactive oxygen species, and it can be oxidized to form GSSG [50, 51]. In this study, significantly improved GSH-Px converted GSSG into GSH to enhance antioxidant status. In addition, SOD and APOA4 genes were notably identified to be upregulated in this study, though the protein products remained unchanged. They may also contribute to the antioxidation of muscle, given the known functions of SOD and the known APOA4 [52]. In the present study, the gene expression variation of superoxide dismutase was consistent with the increasing trend of SOD enzyme activity in the breast muscle in the moderate (0.9 g/kg) EPE treatment groups compared to the control group. This result suggests that EPE improved the activity of SOD, but supplementation with EPE over a long period of time or an increased dose of EPE in chicken feed also substantially impacted the animal's antioxidant capacity. Previous research indicated that the expressions of GST, Prdx6, PPIA, alfatoxin aldehyde reductase, and SOD regulated by albusin B were enhanced to activate the systemic antioxidant defense [16], and SOD, catalase (CAT), GST, and GSH-Px were considered as AOE to protect against oxidative stress [53]. Overall, the functions of these upregulated genes were highly correlated with antioxidant status and could be linked to the improvement of antioxidant capacity induced by EPE supplements.

**Figure 7:**
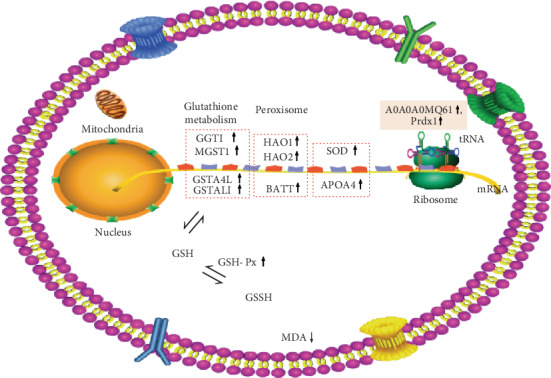
Potential antioxidant mechanism in muscle regulated by EPE.

Many previous studies reported that glutathione is associated with glutathione metabolism pathways and that it likely functions as a reducing milieu to improve antioxidant function in cells [54]. As such, the results of the proteomic analysis indicated that antioxidant-related glutathione transferase activity was significantly increased in response to EPE supplements. The corresponding protein was a kind of glutathione S-transferase (A0A0A0MQ61, EC2.5.1.18) that is known to function in glutathione metabolism in *Gallus gallus.* Additionally, A0A0A0MQ61, an antioxidant protein, was significantly increased as a result of EPE dietary supplements. Further, the upregulated peroxiredoxin-1 protein in the peroxisome metabolic pathway is a known redox-regulating protein and is considered to be an antioxidant enzyme to eliminate various ROS [55]. Based on the above analyses, the antioxidant mechanism of muscle tissue in chicken treated with EPE dietary supplements is inferred and given in Figure 7. As shown, EPE treatments significantly improved GSH-Px activity and decreased MDA content of the breast muscle. Transcriptomic and proteomic analyses revealed that nine candidate genes and two candidate proteins were identified, which are responsible for improving antioxidant status induced by EPE supplements. Overall, this study promotes the understanding of the antioxidant mechanism in chicken regulated by EPE treatment.

## 5. Conclusion

Overall, EPE dietary supplements appeared to improve the antioxidant status of chicken by enhancing GSH-Px activity and reducing MDA content in muscle tissues. Glutathione metabolism and the peroxisome were considered the key metabolic pathways underlying the upregulation of AOE. Furthermore, all of the changes induced by EPE supplements may contribute to the systemic antioxidant defense of chicken, and these altered proteins and genes should be further explored in the future.

## Figures and Tables

**Table 1:** Ingredients and nutrient composition of the experimental diets.

Ingredients (%)	Content	Nutrient level	Content
Corn	64	Metabolic energy (mcal∕kg)	2.96
Wheat shorts	2	Crude protein (%)	15.5
Rice bran	3	Lysine (%)	0.65
Bean pulp	9.7	Calcium (%)	1.19
Peanut bran	4	Phosphorus (%)	0.57
Corn gluten meal	2	Sodium (%)	0.206
Refined three worm powder CP45%	6	Chlorine (%)	0.196
Rock flour	1.5	Potassium (%)	0.47
Calcium hydrophosphate	1.3	Methionine (%)	0.25
Soybean oil	2.5	Arginine (%)	0.83
338 gunk	4		
Total	100		

Note: the vitamin/mineral premix includes (per kg feed): vitamin A, 15750 IU; vitamin D, 3500 IU; vitamin E 35 mg; Menadione, 4.4 mg; Thiamine, 3.5 mg; Riboflavin, 10.5 mg; vitamin B_6_, 7 mg; vitamin B_12_, 35 mg; Nicotinic acid, 70 mg; Pantothenic acid, 21 mg; Folic acid, 1.75 mg; Biotin, 0.175 mg.

**Table 2:** Effect of EPE dietary supplements on antioxidant capacity of serum (*n* = 12).

Item	Control group	0.6 g/kg EPE group	0.9 g/kg EPE group	1.2 g/kg EPE group
T-SOD (U/mgprot)	69.03 ± 1.43	69.23 ± 1.63	70.99 ± 1.96	69.48 ± 1.70
T-AOC (U/mgprot)	11.43 ± 0.66	11.44 ± 0.57	11.56 ± 0.46	11.81 ± 0.63
GSH-Px (U/mgprot)	775.61 ± 45.28	803.12 ± 38.08	815.64 ± 25.76	882.27 ± 57.32
MDA (nmol/mL)	2.45 ± 0.20	2.13 ± 0.28	1.96 ± 0.25	2.12 ± 0.21

**Table 3:** Effect of EPE dietary supplements on antioxidant capacity of breast muscle (*n* = 12).

Item	Control group	0.6 g/kg EPE group	0.9 g/kg EPE group	1.2 g/kg EPE group
T-SOD (U/mgprot)	28.31 ± 5.60	27.48 ± 5.63	33.39 ± 7.82	30.12 ± 4.41
T-AOC (U/mgprot)	7.43 ± 0.81	7.42 ± 1.45	8.39 ± 0.98	8.96 ± 1.75
GSH-Px (U/mgprot)	127.77 ± 24.61^b^	133.64 ± 30.06^ab^	184.63 ± 38.69^a^	159.26 ± 33.58^ab^
GSH (mg/gprot)	3.47 ± 0.25^b^	3.73 ± 0.21^ab^	4.28 ± 0.31^a^	4.19 ± 0.13^a^
MDA (nmol/mgprot)	13.74 ± 1.79^b^	11.26 ± 0.93^ab^	10.25 ± 1.81^a^	11.65 ± 1.50^ab^

^a-b^Means within a row with different superscripts differ significantly (*p* < 0.05).

**Table 4:** GO enrichment analysis for DEGs related antioxidant capacity.

GO id	Description of GO enrichment	Seq id	*p* value	Regulate	Description	Symbol name
GO:0006749	Glutathione metabolic process	ENSGALG00000006565	<0.01	Up	Gamma-glutamyltransferase 1	GGT1
GO:0006749/GO:0016705	Glutathione metabolic process/glutathione transferase activity	ENSGALG00000013098	<0.01	Up	Microsomal glutathione S-transferase 1	MGST1
		ENSGALG00000016324	<0.01	Up	Glutathione S-transferase alpha 4-like	GSTA4L
		ENSGALG00000038652	<0.01	Up	Glutathione S-transferase class-alpha-like 1	GSTAL1
GO:0005777	Peroxisome	ENSGALG00000008845	<0.01	Up	Hydroxyacid oxidase 1	HAO1
		ENSGALG00000014766	<0.01	Up	Hydroxyacid oxidase 2	HAO2
		ENSGALG00000040619	<0.01	Up	Bile acid-CoA: amino acid N-acyltransferase	BAAT

Note: differentially expressed genes (DEGs). The screening criteria for significantly GO enrichment analysis were *p* < 0.05.

**Table 5:** KEGG enrichment pathways for DEGs associated with antioxidant capacity.

Name of KEGG pathway	KEGG ID	Number of different gene	*p* value	Description	Symbol name
Peroxisome	ko04146	6	<0.01	Solute carrier family 27 member 5, alanine-glyoxylate and serine--pyruvate aminotransferase,	SLC27A5, AGXT, PIPOX, HAO1, HAO2, BAAT
				Peroxisomal sarcosine oxidase, hydroxyacid oxidase 1, hydroxyacid oxidase 2, bile acid-CoA: amino acid N-acyltransferase	
Glutathione metabolism	ko00480	4	<0.05	Gamma-glutamyltranspeptidase 1, microsomal glutathione S-transferase 1, glutathione S-transferase class-alpha, glutathione S-transferase alpha 4	GGT1, MGST1, GSTAL1, GSTA4L

Note: differentially expressed genes (DEGs). The screening criteria for significantly KEGG enrichment pathways were *p* < 0.05. Hydroxyacid oxidase 1 (HAO1); hydroxyacid oxidase 2 (HAO2); bile acid-CoA: amino acid N-acyltransferase (BAAT); microsomal glutathione S-transferase 1 (MGST1); glutathione S-transferase class-alpha (GSTAL1); glutathione S-transferase alpha 4 (GSTA4L).

**Table 6:** Significantly DEPs in the proteomics analysis.

Proteins	Description	Fold change	*p* value	Regulate
Q7LZS1	12K serum protein, beta-2-m cross-reactive (fragment)	0.71	0.04	Down
A0A1D5NXR4	Uncharacterized protein	0.34	0.003	Down
F1P372	Uncharacterized protein	1.24	0.03	Up
F1NG89	Ubiquitin carboxyl-terminal hydrolase 10	1.20	0.02	Up
A0A1D5PFH3	Uncharacterized protein	1.28	0.02	Up
F1NHM9	Phosphoglycerate mutase	0.82	0.02	Down
E1C4V1	ATP synthase-coupling factor 6, mitochondrial	1.20	0.02	Up
A0A0A0MQ61	Uncharacterized protein	1.48	0.03	Up
A7UEB0	Alpha-1-acid glycoprotein	0.69	0.01	Down
Q5ZLR5	Cytochrome b-c1 complex subunit Rieske, mitochondrial	1.25	0.01	Up
P02659	Apovitellenin-1	1.41	0.002	Up
Q5ZHZ0	Spliceosome RNA helicase DDX39B	1.21	0.05	Up
Q91968	Alpha-tropomyosin	1.49	0.04	Up
A0A1D5NZ55	Uncharacterized protein	1.30	0.05	Up

Note: differentially expressed proteins (DEPs). The screening criteria for significantly differentially expressed proteins were *p* < 0.05 and (FC < 0.83 or FC > 1.20).

**Table 7:** KEGG enrichment pathways for DEPs.

Pathway	Pathway definition	Number of proteins	Proteins
ko00190	Oxidative phosphorylation	1	Q5ZLR5 (connectin/fragment)
ko01100	Metabolic pathways	1	
ko04260	Cardiac muscle contraction	1	
ko04932	Nonalcoholic fatty liver disease (NAFLD)	1	
ko05010	Alzheimer's disease	1	
ko05012	Parkinson's disease	1	
ko05016	Huntington's disease	1	
ko00480	Glutathione metabolism	1	A0A0A0MQ61 (uncharacterized protein)
ko05204	Chemical carcinogenesis	1	
ko00980	Metabolism of xenobiotics by cytochrome P450	1	
ko00982	Drug metabolism-cytochrome P450	1	
ko00970	Aminoacyl-tRNA biosynthesis	1	F1P372 (uncharacterized protein)

Note: differentially expressed proteins (DEPs). Q5ZLR5 (cytochrome b-c1 complex subunit Rieske, mitochondrial), A0A0A0MQ61 (uncharacterized protein), F1P372 (uncharacterized protein).

## Data Availability

The data used to support the findings of this study are included within the article and the supplementary information file.

## Abbreviations

EPE: Eucalyptus leaf polyphenols extract
GSH-Px: Glutathione peroxidase
MDA: Malonaldehyde
T-SOD: Total superoxide dismutase
T-AOC: Total antioxidant capacity
DEGs: Differentially expressed genes
DEPs: Differentially expressed proteins
GO: Gene Ontology
KEGG: Kyoto Encyclopedia of Genes and Genomes
GGT1: Gamma-glutamyltransferase 1
MGST1: Microsomal glutathione S-transferase 1
GSTA4L: Glutathione S-transferase alpha 4
GSTAL1: Glutathione S-transferase class-alpha
HAO1: Hydroxyacid oxidase 1
HAO2: Hydroxyacid oxidase 2
BAAT: Bile acid-CoA: amino acid N-acyltransferase
SLC27A5: Solute carrier family 27 member 5
AGXT: Alanine-glyoxylate and serine--pyruvate aminotransferase
PIPOX: Peroxisomal sarcosine oxidase
GGT1: Gamma-glutamyltranspeptidase 1
ROS: Reactive oxygen species
GSH: Glutathione
CAT: Catalase
GST: Glutathione-S-transferase
GPX: Glutathione peroxidase
AOE: Antioxidant enzymes.
